# Association of Rural and Critical Access Hospital Status With Patient Outcomes After Emergency Department Visits Among Medicare Beneficiaries

**DOI:** 10.1001/jamanetworkopen.2021.34980

**Published:** 2021-11-19

**Authors:** Margaret Greenwood-Ericksen, Neil Kamdar, Paul Lin, Naomi George, Larissa Myaskovsky, Cameron Crandall, Nicholas M. Mohr, Keith E. Kocher

**Affiliations:** 1 Department of Emergency Medicine, University of New Mexico, Albuquerque; 2 Department of Psychiatry and Behavioral Sciences, University of New Mexico, Albuquerque; 3 Institute for Healthcare Policy and Innovation, University of Michigan, Ann Arbor; 4 Department of Emergency Medicine, University of Michigan, Ann Arbor; 5 Division of Critical Care, Department of Emergency Medicine, University of New Mexico, Albuquerque; 6 Center for Healthcare Equity in Kidney Disease, Department of Internal Medicine, University of New Mexico Health Sciences Center, Albuquerque; 7 Department of Emergency Medicine, University of Iowa, Iowa City; 8 Department of Anesthesia–Critical Care Medicine, University of Iowa, Iowa City; 9 Department of Learning Health Sciences, University of Michigan, Ann Arbor

## Abstract

**Importance:**

Rural US residents disproportionately rely on emergency departments (ED), yet little is known about patient outcomes after ED visits to rural hospitals or critical access hospitals (CAHs).

**Objective:**

To compare 30-day outcomes after rural vs urban ED visits and in CAHs, a subset of rural hospitals.

**Design, Setting, and Participants:**

This propensity-matched, retrospective cohort study used a 20% sample of national Medicare fee-for-service beneficiaries from January 1, 2011, to October 31, 2015. Rural and urban ED visits were matched on demographics, patient prior use of EDs, comorbidities, and diagnoses. Thirty-day outcomes overall and stratified by 25 common ED diagnoses were evaluated, with similar analysis of CAHs vs non-CAHs. Data were analyzed from February 15, 2020, to May 17, 2021.

**Main Outcomes and Measures:**

The primary outcome was 30-day all-cause mortality. Secondary outcomes were ED revisits with and without hospitalization.

**Results:**

The matched cohort included 473 152 rural and urban Medicare beneficiaries with a mean (SD) age of 75.1 (7.9) years (59.1% and 59.3% women, respectively; 86.9% and 87.1% White, respectively). Medicare beneficiaries at rural vs urban EDs experienced similar all-cause 30-day mortality (3.9% vs 4.1%; effect size, 0.01), ED revisits (18.1% vs 17.8%; effect size, 0.00), and ED revisits with hospitalization (6.0% vs 8.1%; effect size, 0.00). Rural ED visits were associated with more transfer (6.2% vs 2.0%; effect size, 0.22) and fewer hospitalizations (24.7% vs 39.2; effect size, 0.31). Stratified by diagnosis, patients in rural EDs with life-threatening illnesses experienced more transfer with 30-day mortality similar to that of patients in urban EDs. In contrast, mortality differed for patients in rural EDs with symptom-based diagnoses, including chest pain (odds ratio [OR], 1.54 [95% CI, 1.25-1.89]), nausea and vomiting (OR, 1.68 [95% CI, 1.26-2.24), and abdominal pain (OR, 1.73 [95% CI, 1.42-2.10]). All findings were similar for CAHs.

**Conclusions and Relevance:**

The findings of this cohort study of rural ED care suggest that patient mortality for potentially life-threatening conditions is comparable to that in urban settings. Further research is needed to understand the sources of greater rural ED mortality for symptom-based conditions. These findings underscore the importance of ensuring access to treatment of life-threatening conditions at local EDs in rural communities, which are increasingly endangered by hospital closures.

## Key Points

**Question:**

Do 30-day outcomes differ after emergency department (ED) visits in rural vs urban settings and in the subset of rural hospitals classified as critical access?

**Findings:**

In this cohort study of 473 152 matched urban and rural Medicare beneficiaries, risk-adjusted all-cause mortality after rural and urban ED visits was similar, particularly for potentially life-threatening conditions. Critical access hospitals had similar outcomes.

**Meaning:**

These findings underscore the importance of rural and critical access EDs for treatment of life-threatening conditions among Medicare recipients and have important policy implications given the continued increase in rural hospital closures.

## Teaser

This cohort study compares 30-day outcomes among Medicare recipients after rural vs urban emergency department visits and in a subset of rural hospitals classified as critical access using national fee-for-service Medicare data.

## Introduction

Visits to rural and critical access hospital (CAH) emergency departments (EDs) have risen 50% in the US in the last 10 years,^1^ particularly for acute, unscheduled care.^2^ This growth reflects the safety net role of EDs in US rural communities, which disproportionately experience primary care shortages^3,4^ and poor health outcomes.^5^ Ongoing rural hospital and CAH closures^6^ are linked to greater rural patient mortality.^7^ However, the value of rural hospitals—specifically CAHs, a subset of rural hospitals that receive enhanced Medicare reimbursement—is frequently debated, pitting health care costs and falling rural hospital inpatient volumes^8^ against the need for 24/7 emergency care access in rural communities.^9,10^ With EDs increasingly serving as sites of care access for rural communities and the only source of emergency care, hospital closures and loss of ED services has a substantial impact on the health of rural residents.

Previous analyses have found higher mortality for inpatient care at rural hospitals^11^ and CAHs^12,13^ compared with urban hospitals, but these analyses did not include the ED setting, and little is known about patient outcomes tied to ED visits. Low case volumes are implicated in driving higher mortality in rural inpatient care,^14,15^ but this issue may not be relevant to emergency care, with many rural and critical access EDs experiencing relatively high patient volumes.^16^ However, there are specific features that may adversely affect emergency care at rural facilities, including limited and declining inpatient capacity^8^ and ED clinicians with varying expertise in emergency care.^17,18^ Similarly, limited access to local specialty consultation in rural areas, on which emergency care is highly reliant,^19^ contributes to high interfacility transfer rates. Although interhospital transfer could mitigate rural mortality risk, the challenges associated with patient transfer^20^ may contribute to delays to definitive care and ultimately worsen outcomes for rural patients.

We therefore designed a study using national Medicare data to examine patient outcomes after rural ED visits. Our primary objective was to compare 30-day outcomes after rural vs urban ED visits and in a subset of rural hospitals classified as critical access. We also assessed 2 secondary outcomes of 30-day ED revisits with and without hospitalization; these are commonly used to contextualize outcomes after ED visits. To understand differences in treatment and transfer patterns between rural and urban centers, we examined hospitalizations and use of interfacility transfers as contextual findings but not as primary or secondary outcomes. We evaluated these outcomes overall and by condition, focusing on both medical and surgical life-threatening diagnoses and symptom-based conditions commonly cared for in EDs. Last, because CAHs are common in rural communities, governed by specific criteria, the subject of recent policy proposals,^21^ and at increased risk of closure related to financial distress,^22^ we conducted an additional analysis on this subset of rural hospitals. Our findings have important policy implications given the continued increase in rural hospital closures and calls to consider new models of rural care delivery that maintain local access to the ED even if inpatient hospital units must close due to unsustainably low volumes.^23^

## Methods

### Study Design and Setting

We performed a retrospective cohort study of all ED encounters between from January 1, 2011, and October 31, 2015, using a 20% random sample^24^ of US fee-for-service Medicare beneficiaries. Medicare data are essential for understanding the provision of health care for older US adults, who commonly and disproportionately access the ED for care in both rural and urban settings relative to other age groups. The University of Michigan Institutional Review Board approved the study, which did not require informed consent for the use of a limited data set without identifiers. The study followed the Strengthening the Reporting of Observational Studies in Epidemiology (STROBE) reporting guideline for observational studies.^25^

We merged several Medicare files using previously described techniques to create our analytic cohort of ED patient visits, including the Medicare Provider Analysis and Review (MedPAR), Outpatient, Carrier, and Master Beneficiary Summary Files.^26^ We linked these data to the American Hospital Association national survey, a rich repository of standardized descriptive information collected annually about each hospital in the US, including facility structure, organization, work force, and capabilities. This permitted augmentation of information about hospital characteristics, identification of hospital urban or rural designation (in accordance with the American Hospital Association designation), and critical access designation.

### Selection of Participants

**Figure 1.**
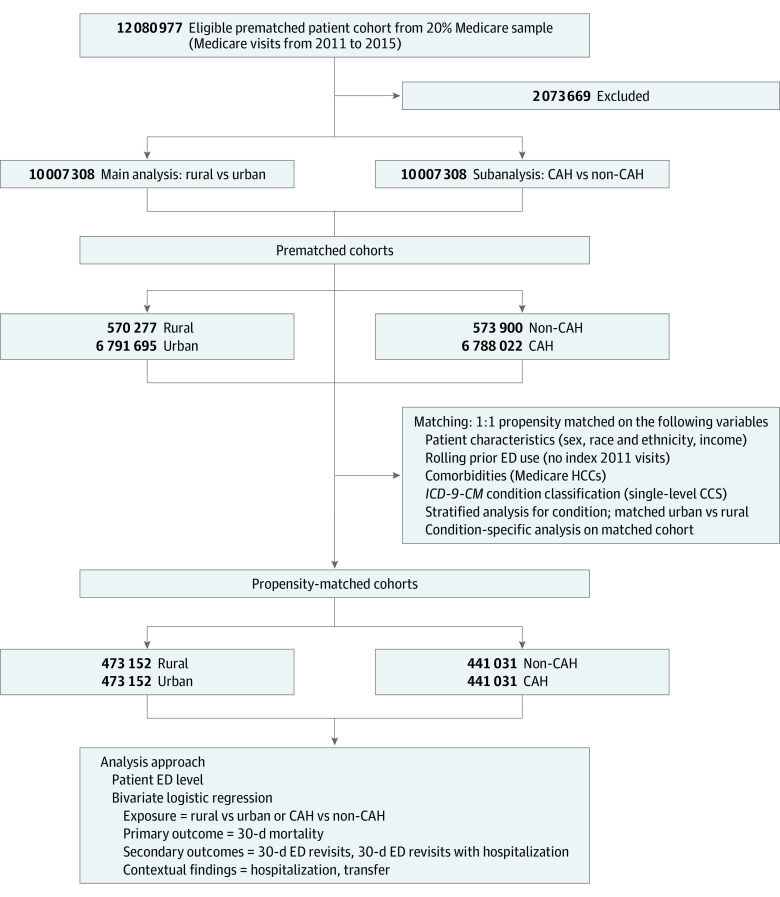
STROBE Diagram for Patient Inclusion Abbreviations: CAH indicates critical access hospital; ED, emergency department; HCC, hierarchical condition category; *ICD-9-CM*, *International Classification of Diseases, Ninth Revision, Clinical Modification.*

The unit of analysis was the patient ED visit encounter. All ED visits between 2011 and 2015 were eligible for our study. We excluded visits in which beneficiaries were younger than 65 years and those who died in the ED, because these events are extremely rare, often do not reflect ED care processes because these patients have little chance of survival, and bias against centers serving populations with high levels of morbidity. We additionally excluded those without 12 months of data before the index ED visit and 30 days of enrollment in Medicare after the index visit, enabling identification of 30-day outcomes as well as a standard period for a patient-specific count of prior ED use (Figure 1).

For 2015, we included claims through October to maintain consistency in use of the *International Classification of Diseases, Ninth Revision, Clinical Modification*, diagnosis codes. After that time, the US transitioned to *International Statistical Classification of Diseases and Related Health Problems, Tenth Revision*, which could have introduced coding and classification differences.^27^ Based on the Clinical Classification Software categories,^28^ we placed ED visits into 25 commonly studied acute conditions^29^ that were mutually exclusive and based on their ED principal diagnosis code (first position diagnosis for each patient’s claim). The ED principal diagnosis code is a clinician-assigned diagnosis and as such is subject to variability rather than reflecting a fixed patient characteristic. These diagnoses are frequently encountered in the ED and represent a range of illness types, including medical and surgical disease (eTable 3 in the Supplement). Some are considered symptom-based (eg, chest pain) and are generally characterized by ambiguity in diagnosis and management. These contrast with specific diagnoses, which are generally definitive with well-established treatment guidelines. Specific diagnoses can be life-threatening (eg, acute coronary syndrome) or more benign (eg, urinary tract infection).

### Measurement Definitions

#### Patient Visit Characteristics

Patient visit characteristics included age, sex, race and ethnicity, ZIP code–level income, and comorbidities (defined as a count of Medicare’s hierarchical condition categories [HCCs]).^30^ To account for prior use of health care services, we created a patient-specific count of the number of ED visits in the 12 months before their index ED visit. We also assessed hospital characteristics (ED volume/size, region, ownership) to facilitate interpretation. Except for teaching status, these were not included in the modeling, given inadequate cases per hospital to include hospital-level effects.

#### Index ED Visit

We defined an index ED visit as the patient’s contact with the first hospital to deliver care even if subsequently transferred.^31^ Patient visit outcomes were therefore attributed to that original facility, avoiding misclassification bias. We created an indicator for hospitalization, transfer, and discharge for each ED encounter. We defined hospitalizations as ED visits ending in either a standard inpatient admission, placement in observation status, or interhospital transfer. We chose this broad definition of hospitalization because transfer is often intended to result in hospital admission. Transfers were also considered separately, defined as an ED visit that resulted in transfer to another acute care hospital. Given that this is a national, standardized data set, we have no reason to suspect systemic misclassification bias of transfers within the available administrative claims information. Emergency department visits were defined as discharges if representing treat-and-release encounters.

#### Outcomes

The primary outcome was 30-day mortality, which we defined as death due to any cause and at any time during the hospital stay and within 30 days of hospital discharge as listed in the Social Security Death Master File. Similar to previous studies, we did not stratify visit mortality by disposition (eg, hospitalized vs discharged).^32^ Secondary outcomes were 30-day ED revisits (return ED visit to any facility within 30 days after the index ED encounter) overall and those that resulted in hospitalization. We evaluated these outcomes overall and by each of the 25 conditions. These 25 conditions were examined for exploratory rather than causal purposes to identify whether some were more susceptible to worse outcomes in the rural setting.

### Statistical Analysis

Data were analyzed from February 15, 2020, to May 17, 2021. We first compared hospital and patient visit characteristics for rural vs urban hospitals and CAHs vs non-CAHs using χ^2^ tests and effect size (Cohen d) calculations with standardized differences. Given the frequency of ED visits within a national Medicare population, our study sample size was very large (Figure 1). Such analyses are statistically overpowered to detect differences in means or proportions. Thus, we calculated Cohen d and h statistics on numerical and categorical variables, respectively, for effect size calculations to obtain clinically meaningful differences for prematch and postmatch comparisons as well as for overall outcome measures.^33^

We then performed a 1:1 propensity match without replacement using a caliper size of 0.0001 for rural and urban ED visits, matching on year, patient visit demographics (sex, race and ethnicity, ZIP code–level income), comorbidities (count of Medicare HCCs), number of prior ED visits, and 25 diagnostic groups for ED visit acute illness. Hierarchical condition category coding is a risk-adjustment model originally designed by the Centers for Medicare & Medicaid Services to estimate complexity of patients and thus a reflection of chronic disease burden.^30^ We then repeated the same process for CAH and non-CAH ED visits. Within all variables, the standardized differences for each matched covariate were less than 0.1, indicating covariates were well matched.

Using the propensity-matched cohort, we conducted bivariate logistic regression on the overall cohort and for the stratified analysis of each of the 25 acute illness conditions using rural vs urban (and CAH vs non-CAH) ED encounters as exposure variables. We then estimated odds ratios (ORs) and marginal effects (MEs) with 95% CIs for each of the outcomes for the overall cohort and for the subsets with the clinical conditions. All 25 clinical conditions studied had sufficiently large sample sizes (eTable 3 in the Supplement) such that OR estimation in the postmatched cohort was reliable. Thus, we used effect size to measure meaningful differences in overall cohort outcomes due to very large sample sizes and 95% CI for stratified outcomes given sufficiently smaller sample sizes. All analyses were performed with SAS, version 9.4 (SAS Institute Inc). Two-sided *P* < .05 indicated statistical significance.

## Results

### Characteristics of Study Subjects

Our prematch sample was composed of 570 277 patient visits at rural EDs and 6 791 695 patient visits at urban EDs, with 473 152 in each group after matching (rural group, 59.1% women and 40.9% men; urban group, 59.3% women and 40.7% men; mean [SD] age for both groups, 75.1 [7.9] years). Unmatched visit characteristics demonstrated that Medicare beneficiaries seeking care at rural hospitals and CAHs included more White patients (88.7% vs 81.5%; effect size, 0.20) with lower mean (SD) incomes ($41 917 [$12 165] vs $56 913 [$22 782]; effect size, 0.70) than their counterparts, but a similar comorbidity burden as reflected by Medicare HCC (eg, for category 3, 12.8% vs 14.7%; effect size, 0.06) (Table 1). The matched cohort included 473 152 rural and urban Medicare beneficiaries with a mean (SD) age of 75.1 (7.9) years (59.1% and 59.3% women, respectively; 40.9% and 40.7% men, respectively; 86.9% and 87.1% White, respectively). We found similar results in our analysis of CAH vs non-CAH EDs (eTable 1 in the Supplement).

**Table 1.** Characteristics of Patient Visits Treated at Urban and Rural Hospitals Before and After Propensity-Score Matching

Characteristic	Unmatched patients			Propensity-score–matched patients		
	Hospital setting^a^		Effect size^b^	Hospital setting^a^		Effect size^b^
	Urban (n = 6 791 695)	Rural (n = 570 277)		Urban (n = 473 152)	Rural (n = 473 152)	
Age, mean (SD), y	75.4 (8.0)	75.0 (7.8)	0.05	75.1 (7.9)	75.1 (7.9)	0.001
Sex						
Female	4 070 337 (59.9)	333 647 (58.5)	0.03	280 463 (59.3)	279 446 (59.1)	0.001
Male	2 721 358 (40.1)	236 580 (41.5)	0.02	192 689 (40.7)	193 706 (40.9)	0.001
Race and ethnicity						
Black	647 770 (9.5)	37 882 (6.6)	0.11	34 924 (7.4)	35 675 (7.5)	0.01
White	5 539 020 (81.5)	505 619 (88.7)	0.20	412 297 (87.1)	411 252 (86.9)	0.01
Other	604 905 (8.9)	26 726 (4.7)	0.17	25 931 (5.5)	26 225 (5.5)	0.001
Medicare HCC^c^						
1	3 930 219 (57.9)	337 033 (59.1)	0.03	271 146 (57)	280 951 (59.4)	0.04
2	1 860 831 (27.4)	160 471 (28.1)	0.02	132 206 (28)	132 512 (28.0)	0.001
3	1 000 645 (14.7)	72 723 (12.8)	0.06	10 297 (15)	59 689 (12.6)	0.06
Household income, median (SD), $	56 913 (22 782)	41 917 (12 165)	0.68	43 744 (12 023)	43 526 (12 395)	0.03

Abbreviation: HCC, hierarchical condition category.

^a^ Unless otherwise indicated, data are expressed as number (%) of patients. Percentages have been rounded and may not total 100.

^b^ All differences were calculated as Cohen d or h statistics to assess meaningful differences and represent a standardized mean difference in proportions for categorical variables or means for continuous variables.

^c^ Indicates comorbidity categories in ascending order of medical complexity (eg, HCC category 1 is the least complex; HCC category 3 is most complex).

Structural differences existed between hospitals. Rural hospitals and CAHs were more likely to be government owned (496 of 1127 [42.5%] vs 546 of 3410 [16.0%]; *P* < .001), with fewer inpatient beds (<100, 1075 of 1167 [92.1%] vs 1225 of 3410 [35.9%]; *P* < .001), and less likely to be in the Northeast (53 of 1148 [4.6%] vs 512 of 3336 [15.3%]; *P* < .001) compared with urban hospitals (Table 2 and eTable 2 in the Supplement). In our sample, 607 rural hospitals (52.0%) also had a CAH designation. For the condition-stratified analysis, our prematched data indicated that rural and urban EDs had similar counts of diagnoses for all conditions (eTable 3 in the Supplement).

**Table 2.** Characteristics of Hospitals Treating Patients by Urban and Rural Status, Before Propensity Matching

Characteristic	Unmatched hospitals, No. (%)		*P* value
	Urban	Rural	
Hospitals	3410 (74.5)	1167 (25.5)	<.001
Hospital ownership			
For-profit	684 (20.1)	108 (9.3)	<.001
Nonprofit	2180 (63.9)	563 (48.2)	<.001
Government	546 (16.0)	496 (42.5)	<.001
No. of hospital beds			
<100	1225 (35.9)	1075 (92.1)	<.001
100-199	895 (26.2)	84 (7.2)	<.001
200-499	1015 (29.8)	8 (0.7)	<.001
≥500	275 (8.1)	0	<.001
Census region^a^			
Northeast	512 (15.3)	53 (4.6)	<.001
Midwest	892 (26.7)	463 (40.3)	<.001
South	1274 (38.2)	459 (40.0)	<.001
West	658 (19.7)	173 (15.1)	<.001
Teaching status			
No residents	2198 (64.5)	1085 (93.0)	<.001
Minor (<0.25 residents/bed)	974 (28.6)	79 (6.8)	<.001
Major (≥0.25 residents/bed)	237 (7.0)	3 (0.3)	<.001

^a^ Defined according to US Census regions.

### Main Results

After propensity matching, Medicare beneficiaries treated in rural compared with urban EDs had similar all-cause 30-day mortality (3.9% vs 4.1%; effect size, 0.01), ED revisits (18.1% vs 17.8%; effect size, 0.00), and ED revisits with hospitalization (6.0% vs 8.1%; effect size, 0.00). Rural beneficiaries experienced a lower proportion of hospitalization than those seen in urban EDs (24.7% vs 39.2; effect size, 0.31) and a greater proportion of interhospital transfer (6.2% vs 2.0%; effect size, 0.22).

**Figure 2.**
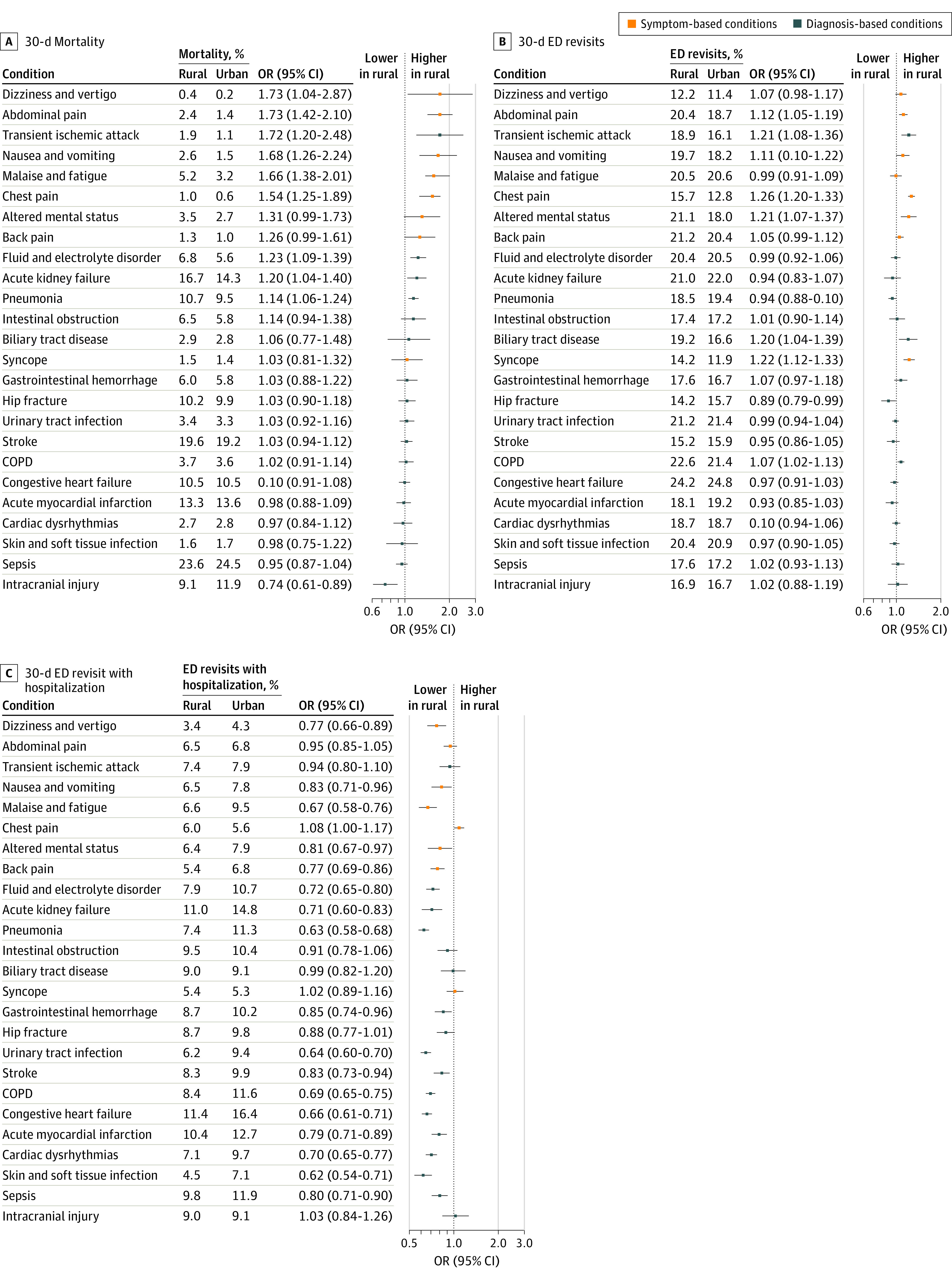
Rural vs Urban Mortality and Emergency Department (ED) Revisits by Commonly Encountered ED Conditions Conditions are sorted by mortality outcome. COPD indicates chronic obstructive pulmonary disorder. Abbreviation: OR indicates odds ratio.

**Figure 3.**
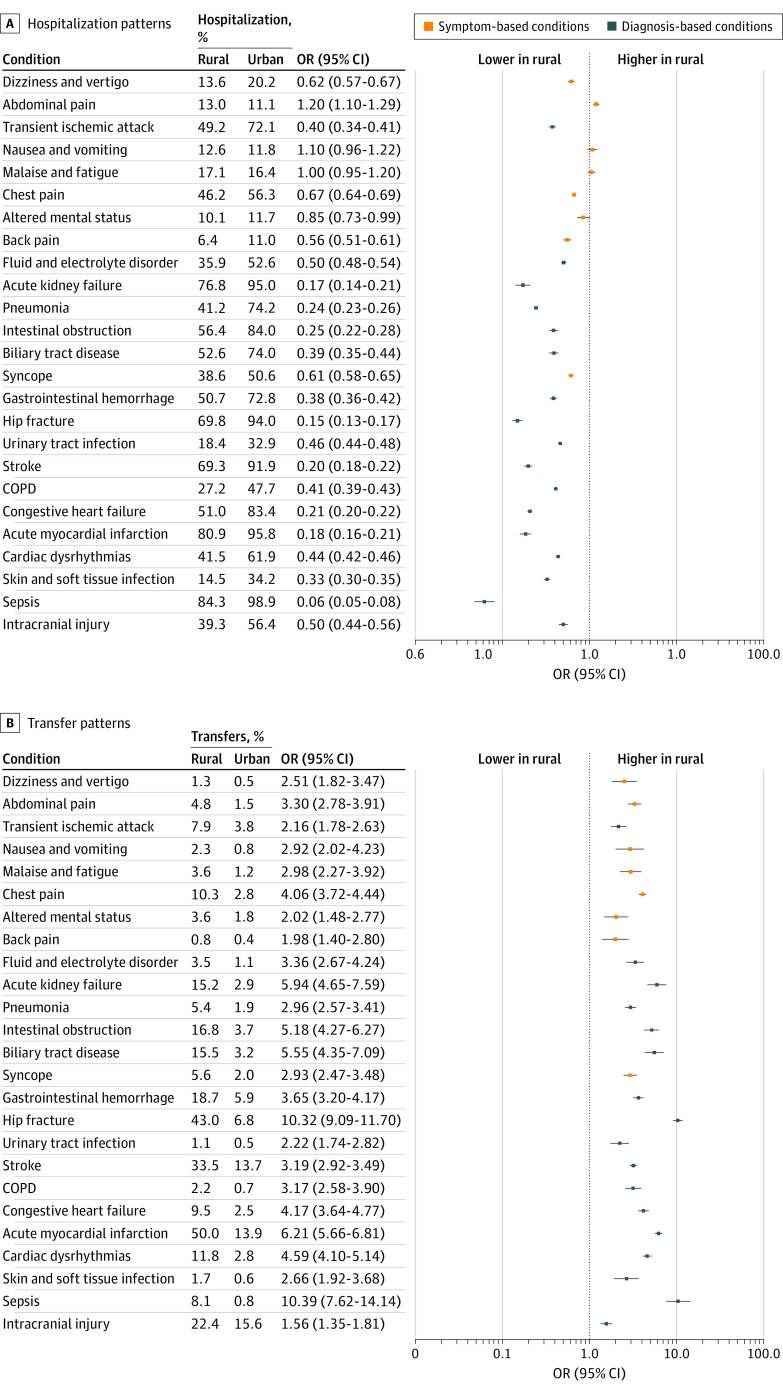
Rural vs Urban Hospitalization and Transfer Patterns by Commonly Encountered Emergency Department (ED) Condition Conditions sorted by mortality outcome as shown in Figure 2. Abbreviations: COPD indicates chronic obstructive pulmonary disorder; OR, odds ratio.

Stratification by diagnosis demonstrated differences between conditions in primary and secondary outcomes. Rural patient mortality was similar for life-threatening diagnoses such as sepsis (OR, 0.95 [95% CI, 0.87-1.04]; marginal effects [ME], 0.9% [95% CI, −0.7 to 2.5]), stroke (OR, 1.03 [95% CI, 0.94-1.12]; ME, −0.4% [95% CI, −1.8 to 0.6]), and myocardial infarction (OR, 0.98 [95% CI, 0.88-1.09]; ME, 0.2% [95% CI, −1.0 to 1.5]) but was greater for symptom-based diagnoses such as chest pain (OR, 1.54 [95% CI, 1.25-1.89]; ME, −0.3% [95% CI, −0.5 to −0.2]), malaise and fatigue (OR, 1.66 [95% CI, 1.38-2.01]; ME, −2.0 [95% CI, −2.7 to −1.3]), nausea and vomiting (OR, 1.68 [95% CI, 1.26-2.24]; ME, −1.0 [95% CI, −1.6 to −0.5]), and abdominal pain (OR, 1.73 [95% CI, 1.42-2.10]; ME, −1.0% [95% CI, −1.3 to −0.6]) (Figure 2 and eTable 5 in the Supplement). For the secondary outcome of 30-day ED return visits, rural patients were similarly likely to experience these events compared with urban patients across the diagnoses studied. However, they were less likely to be hospitalized during those return visits (Figure 3). Comparison of CAH and non-CAH facilities demonstrated similar findings (eFigure 1 and eFigure 2 in the Supplement).

## Discussion

Our study is the first, to our knowledge, to systematically examine national patient outcomes overall and by common conditions after ED visits to rural hospitals and CAHs. In general, we found similar 30-day all-cause mortality and ED revisits with and without hospitalization for patients seeking care at rural and urban EDs. Rural ED practice incorporated fewer hospitalizations and more transfers than urban ED practice. When stratified by condition, we found no difference in mortality for patients treated at rural vs urban EDs for potentially life-threatening diagnoses. These findings underscore the importance of ensuring access to treatment for life-threatening illness at EDs in rural communities, which are increasingly experiencing hospital closures.

This analysis presents important foundational exploratory work in understanding the landscape of rural ED care delivery. Our results suggest the rural ED system functions well for discrete conditions with highly codified diagnostic approaches, treatments, and transfer pathways. However, our data also revealed mortality differences among patients presenting to rural hospitals with symptom-based diagnoses, which are generally characterized by ambiguity in diagnosis and management. It is possible that unmeasured clinical or contextual factors may contribute to the risk of worse outcomes for symptom-based conditions in the rural setting. To date, no prior work has drawn comparisons; this national, high-level overview sets the foundation for future research on root causes of these potential outcome differences.

We found no mortality differences between urban and rural ED–treated patients for potentially life-threatening conditions (eg, stroke, sepsis, myocardial infarction). This may be explained by the relatively standardized management algorithms and transfer pathways for these conditions once diagnosed. The literature examining rural ED–based care quality for life-threatening illness is limited and shows mixed findings. For example, rural EDs perform well on quality of sepsis care,^34^ but patients with stroke at rural facilities are less likely to receive thrombolytics^35^ and have higher mortality.^36^ Factors thought to play a role in outcomes at rural hospital EDs include rural hospital bypass, with sicker patients transported by emergency medical services to larger hospitals, as in the case of trauma (and likely reflected in lower odds of mortality for rural patients with cerebral injury in our study)^37^ and patient-initiated bypass,^38,39^ although the latter has been linked to greater mortality in sepsis.^40^ Our data suggest that the complex system of interfacility transfer that supports rural hospitals helps to ensure similar outcomes for people experiencing life-threatening illness at rural EDs. Efforts to improve interfacility transfer for select life-threatening conditions^41,42^ through regionalization^37,43,44,45^ and standardization of processes^46^ have reduced mortality and improved quality measure performance. Although our data may suggest that interfacility transfer is optimized, it is often described as difficult, deeply fragmented,^20^ and requiring complex coordination efforts by the rural clinician.^47^ Rural policy and health care advocates should continue to focus on improving interfacility transfer systems for rural patients.

Our data show that patients with several symptom-based conditions experience greater mortality after rural and CAH ED visits. These differences may be associated with patient-, clinician-, hospital-, and system-level factors. First, there may be unmeasured clinical and contextual (eg, sociocultural) patient factors. Rural persons may be at risk for worse outcomes given higher rates of poorly controlled chronic disease,^48,49^ obesity,^50^ and smoking.^51^ In addition, rural patients may present in a more advanced stage of acute illness owing to geographic distances and be less desirous of hospitalization (and transfer) if they are far from home. Second, although no literature exists on this topic, it is possible that misdiagnosis or inferior care may be relatively more common in rural ED settings and could influence outcomes. We found in our prematch data that rural and urban clinicians diagnosed symptom-based conditions proportionally (eTable 3 in the Supplement), which is reassuring against misdiagnosis, although it cannot fully eliminate the concern of care differences contributing to worse outcomes. Third and potentially related, hospitalization, which is less common for rural compared with urban patients in all conditions studied, may mitigate short-term risk of mortality from symptom-based conditions—particularly in the setting of limited outpatient follow-up. Both hospitalization and transfer yield the benefit of another clinician’s evaluation, monitoring, and observation of the evolution of illness. However, the mitigating effect of hospitalization on mortality varies by condition. For example, hospitalization may reduce short-term risk of death in certain types of pneumonia, but may not be as effective in a condition such as syncope, in which hospitalization yields minimal diagnostic and therapeutic benefit.^52^ A previous study has also demonstrated greater risk of mortality after an ED visit with lower-than-average hospitalization rates, particularly for symptom-based conditions.^53^ Although our study was not designed to examine hospitalization as an outcome measure, our findings of lower odds of hospitalization suggest that these practices may have moderating effects on mortality.

The root causes of the 30-day mortality difference finding cannot be determined from this study but may be linked to rural-specific challenges. Potential barriers to hospitalization after a rural ED visit may include patient preference, clinician decision-making, and limited inpatient capacity of rural hospitals. Further, the experience of rural ED clinicians, which varies across rural hospitals and CAHs, accompanied by lack of consistent access to specialty consultation and diagnostic resources (eg, magnetic resonance imaging) may play a role. Patient-level factors such as average older age^54^ and a greater burden of comorbidities (eg, diabetes^48^ and obesity^50^) in rural communities may also affect these outcomes. However, in our unmatched sample, rural and urban patients had similar HCCs, suggesting that individuals who live in rural settings are not significantly more medically complex. Finally, symptom-based conditions may be susceptible to poor outcomes owing to limited rural primary care access after an ED visit.^55,56^ Although no literature exists on the interplay between clinician decision-making regarding hospitalization and hospitalization preference by patients in rural vs urban communities, this unmeasurable factor may contribute to these findings.

As emergency care practices advance, evidence suggests that rural hospitals may not be achieving the same gains as their urban counterparts. For example, our study’s disparate outcomes between rural patients who are transferred away from rural hospitals and those who are hospitalized or discharged locally may represent a mechanism for the smaller associated mortality reduction over time by rural EDs, as noted in a recent study.^32^ These findings may reflect lack of access to technology, specialists,^19^ structural factors (eg, staffing), or low patient volume effects^29^; ultimately, they raise concerns that improvements in urban emergency care delivery are not equally realized in rural settings, which may require additional support and resources.

### Limitations

This study has several limitations. Although administrative data offer large sample sizes and accurate longitudinal follow-up, billing codes can be imperfect in discriminating between patient illness severity. Propensity score matching does not fully account for latent or unobserved confounding. Furthermore, our sample of older US residents in a federal insurance program is highly relevant to rural health but may not be generalizable to other populations with different insurance coverage or demographics. Although CAH coding practices may result in undercoding of comorbidities using HCCs,^57^ this would not be the case at rural non-CAH EDs where findings were similar. Future work should include measures of clinical severity to determine illness severity at presentation. Finally, we do not know whether the outcomes reflect rural ED care or selection bias, nor could we identify the root cause of the differences in outcomes between diagnosis types. Although covariate balance holds for overall comparisons (eTable 4 in the Supplement), the 25 conditions studied are highly heterogenous owing to variable approaches to diagnosis, severity, and treatment; therefore, their outcomes are not directly comparable.

## Conclusions

The findings of this study underscore the importance of ensuring access to local EDs in rural communities, which are endangered by increasing rural hospital closures. Although our findings should be tempered with the limitations of our analysis, this work should also inform health system leaders and policy makers on the valuable role of ED care in facilitating similar outcomes for individuals in rural settings who have life-threatening illnesses. Our findings primarily represent important foundational exploratory work, and future research should explore sources of the mortality differences in symptom-based conditions.

## Notes

Supplement. **eTable 1.** Characteristics of Patient Visits Treated at Non-CAHs and CAHs Before and After Propensity Score Matching **eTable 2.** Characteristics of Hospitals Treating Patients by Non-CAH and CAH Status Before Propensity Matching **eTable 3.** Patient Counts and Percentages by Diagnosis (*ICD-9-CM*) **eTable 4.** Propensity Scores for Each Decile of Rural and Urban Patients **eTable 5.** Marginal Effects with 95% CI of 30-Day Mortality for Urban vs Rural and Non-CAH vs CAH **eFigure 1.** Critical Access vs Non–Critical Access Mortality and ED Revisits by Commonly Encountered ED Conditions **eFigure 2.** Critical Access vs Non–Critical Access Hospitalization and Transfer Patterns by Commonly Encountered ED Condition
