# Supplementary material for: Association of Rural and Critical Access Hospital Status With Patient Outcomes After Emergency Department Visits Among Medicare Beneficiaries
Source: JAMA Netw Open. 2021 Nov 19;4(11):e2134980. doi: 10.1001/jamanetworkopen.2021.34980 (PMC8605483; doi:10.1001/jamanetworkopen.2021.34980)
Supplement: Supplement. — eTable 1. Characteristics of Patient Visits Treated at Non-CAHs and CAHs Before and After Propensity Score Matching eTable 2. Characteristics of Hospitals Treating Patients by Non-CAH and CAH Status Before Propensity Matching eTable 3. Patient Counts and Percentages by Diagnosis (ICD-9-CM) eTable 4. Propensity Scores for Each Decile of Rural and Urban Patients eTable 5. Marginal Effects with 95% CI of 30-Day Mortality for Urban vs Rural and Non-CAH vs CAH eFigure 1. Critical Access vs Non–Critical Access Mortality and ED Revisits by Commonly Encountered ED Conditions eFigure 2. Critical Access vs Non–Critical Access Hospitalization and Transfer Patterns by Commonly Encountered ED Condition [file jamanetwopen-e2134980-s001.pdf]

Supplementary Online Content

Greenwood-Ericksen M, Kamdar N, Lin P, et al. Association of rural and critical access hospital status with patient outcomes after emergency department visits among Medicare beneficiaries. *JAMA Netw Open*. 2021;4(11):e2134980. doi:10.1001/jamanetworkopen.2021.34980

eTable 1. Characteristics of Patient Visits Treated at Non-CAHs and CAHs Before and After Propensity Score Matching
eTable 2. Characteristics of Hospitals Treating Patients by Non-CAH and CAH Status Before Propensity Matching
eTable 3. Patient Counts and Percentages by Diagnosis (*ICD-9-CM*)
eTable 4. Propensity Scores for Each Decile of Rural and Urban Patients
eTable 5. Marginal Effects with 95% CI of 30-Day Mortality for Urban vs Rural and Non-CAH vs CAH
eFigure 1. Critical Access vs Non–Critical Access Mortality and ED Revisits by Commonly Encountered ED Conditions
eFigure 2. Critical Access vs Non–Critical Access Hospitalization and Transfer Patterns by Commonly Encountered ED Condition

This supplementary material has been provided by the authors to give readers additional information about their work.

© 2021 Greenwood-Ericksen M et al. *JAMA Network Open*.

eTable 1. Characteristics of Patient Visits Treated at Non-CAHs and CAHs Before and After Propensity Score Matching

**Characteristic**	**Unmatched Patients**			**Propensity-Score–Matched Patients**		
	Non-CAH (N = 573,900)	CAH (N = 6,788,022)	Effect Size	Non-CAH (N = 441,031)	CAH (N = 441,031)	Effect Size
Age, mean (SD)	75.4 (8.0)	75.2 (7.9)	0.03	75.3 (7.9)	75.1 (7.9)	0.01
Female, no. (%)	4,070,137 (60%)	573,900 (58%)	0.04	291,086 (59%)	289,131 (59)	0.01
Race/ethnicity, no. (%)						
White	5,515,305 (81%)	527,334 (92%)	0.30	492,608 (91%)	446,302 (91%)	0.02
Black	655,822 (10%)	19,770 (3%)	0.30	17,442 (4%)	19,764 (4%)	0.02
Other	604,835 (10%)	26,796 (5%)	0.20	25,832 (5%)	26,542 (5%)	0.01
HCC Count, no. (%)						
Category 1	3,916,804 (58%)	350,448 (61%)	0.07	283,180 (57%)	302,213 (61%)	0.08
Category 2	6,788,022 (27%)	155,647 (27%)	0.01	138,265 (28%)	132,931 (27%)	0.02
Category 3	6,788,022 (15%)	67,805 (12%)	0.09	71,163 (14%)	57,464 (12%)	0.08
Income, median (SD)	56,647 (22,918)	45,154 (12,172)	0.52	46,419 (13,438)	46,227 (13,203)	0.01

Legend: SD = Standard deviation; Income: median household income**;** HCC Count: comorbidity count in ascending order of complexity (e.g., HCC category 1 is the least complex; HCC category 3 is most complex). Effect size: all differences were calculated as Cohen's d or h statistics to assess meaningful differences and represent a standardized mean difference in proportions for categorical variables or means for continuous variables. This table is identical format to manuscript's **Table 1** for ease of comparison between rural vs. urban and CAH vs. non-CAH.

© 2021 Greenwood-Ericksen M et al. *JAMA Network Open*.

eTable 2. Characteristics of Hospitals Treating Patients by Non-CAH and CAH Status Before Propensity Matching

Characteristic	Unmatched Hospitals		
	Non-CAH	CAH	P-value
Hospitals, no. (%)			
Hospital ownership			
For-profit	721 (22.2)	71 (5.3)	<.0001
Nonprofit	2023 (62.3)	720 (54.2)	<.0001
Government	504 (15.5)	538 (40.5)	<.0001
Hospital Bed, no. (%)			
<100	1027 (31.6)	1273 (95.8)	<.0001
100-199	925 (28.5)	54 (4.2)	<.0001
200-499	1021 (31.4)	2 (0.2)	<.0001
<500	275 (8.5)	0 (0.00)	<.0001
Census region, no (%)			
Northeast	494 (15.6)	71 (5.4)	<.0001
Midwest	723 (22.8)	632 (48.3)	<.0001
South	1384 (43.6)	349 (26.7)	<.0001
West	575 (18.1)	256 (19.6)	<.0001
Teaching Status, no (%)			
No residents	1997 (61.5)	1286 (96.8)	<.0001
Minor (<0.25 residents/bed)	1010 (31.1)	43 (3.2)	<.0001
Major > (0.25 residents/bed)	240 (7.4)	0 (0.00)	<.0001

Legend: This table reports pre-matched data to provide the reader a comparison between the types of hospitals. Census region defined by U.S. census regions. This table is identical format to manuscript's **Table 2** for ease of comparison between rural vs. urban and CAH vs. non-CAH.

© 2021 Greenwood-Ericksen M et al. *JAMA Network Open*.

**eTable 3.** Patient Counts and Percentages by Diagnosis (*ICD-9-CM*)				
**Diagnosis**	**Overall (count, %)**	**Urban (count, %)**	**Rural (count, %)**	**Effect Size**
Chest pain	446124 (4.49)	409615 (4.47)	36509 (4.77)	0.014
Urinary Tract Infection	351108 (3.54)	319973 (3.49)	31135 (4.06)	0.030
Chronic obstructive pulmonary disease	294936 (2.97)	263527 (2.88)	31409 (4.10)	0.067
Cardiac dysrhythmia	307201 (3.09)	286120 (3.12)	21081 (2.75)	0.022
Pneumonia	256252 (2.58)	230979 (2.52)	25273 (3.30)	0.047
Congestive heart failure	285427 (2.88)	265291 (2.90)	20136 (2.63)	0.016
Abdominal pain	239204 (2.41)	219262 (2.39)	19942 (2.60)	0.013
Syncope	185575 (1.87)	172021 (1.88)	13554 (1.77)	0.008
Septicemia	272693 (2.75)	264673 (2.89)	8020 (1.05)	0.136
Cerebrovascular accident (e.g. stroke)	161623 (1.63)	153268 (1.67)	8355 (1.09)	0.050
GI hemorrhage	120468 (1.21)	111928 (1.22)	8540 (1.11)	0.010
Acute myocardial infarction	125728 (1.27)	118093 (1.29)	7635 (1.00)	0.027
Acute renal failure	119189 (1.20)	114795 (1.25)	4394 (0.57)	0.073
Intestinal obstruction	84016 (0.85)	78254 (0.85)	5762 (0.75)	0.011
Femur fracture	102222 (1.03)	95893 (1.05)	6329 (0.83)	0.023
Fluid/electrolyte disturbance	175972 (1.77)	158927 (1.73)	17045 (2.23)	0.036
Malaise and fatigue	111899 (1.13)	101649 (1.11)	10250 (1.34)	0.021
Transient ischemic attack	80643 (0.81)	74461 (0.81)	6182 (0.81)	0.000
Skin infection	169641 (1.71)	156546 (1.71)	13095 (1.71)	0.000
Dizziness and vertigo	180708 (1.82)	166118 (1.81)	14590 (1.90)	0.007
Nausea and vomiting	92517 (0.93)	83549 (0.91)	8968 (1.17)	0.026
Spondylosis (e.g., back pain)	235752 (2.37)	217052 (2.37)	18700 (2.44)	0.005
Intracranial injury	63345 (0.64)	60121 (0.66)	3224 (0.42)	0.033
Biliary tract disease	62239 (0.63)	58695 (0.64)	3544 (0.46)	0.024

Legend. Effect size: all differences were calculated as Cohen's d or h statistics to assess meaningful differences and represent a standardized mean difference in proportions for categorical variables or means for continuous variables.

© 2021 Greenwood-Ericksen M et al. *JAMA Network Open*.

eTable 4. Propensity Scores for Each Decile of Rural and Urban Patients

**Rural Patient Analysis**					
Propensity Score Estimated Probability					
**Rank for Propensity Score**	**N**	**Mean**	**Std Dev**	**Minimum**	**Maximum**
**0**	47315	0.0227125	0.0117698	7.7386965E-7	0.0415414
**1**	47315	0.0568548	0.0088716	0.0415430	0.0718360
**2**	47315	0.0863763	0.0083504	0.0718361	0.1003536
**3**	47316	0.1121866	0.0065796	0.1003537	0.1233859
**4**	47315	0.1337784	0.0058770	0.1233861	0.1437916
**5**	47315	0.1534328	0.0055166	0.1437922	0.1629547
**6**	47316	0.1731679	0.0060929	0.1629548	0.1841410
**7**	47315	0.1970900	0.0078689	0.1841418	0.2114845
**8**	47315	0.2299156	0.0115178	0.2114847	0.2519950
**9**	47315	0.3038274	0.0494336	0.2519965	0.6840756

**Urban Patient Analysis**					
Propensity Score Estimated Probability					
**Rank for Propensity Score**	**N**	**Mean**	**Std Dev**	**Minimum**	**Maximum**
**0**	47315	0.0227125	0.0117698	7.7457605E-7	0.0415415
**1**	47315	0.0568548	0.0088716	0.0415430	0.0718358
**2**	47315	0.0863763	0.0083504	0.0718359	0.1003538
**3**	47316	0.1121866	0.0065796	0.1003538	0.1233878
**4**	47315	0.1337784	0.0058770	0.1233881	0.1437901
**5**	47315	0.1534327	0.0055165	0.1437904	0.1629486
**6**	47316	0.1731678	0.0060928	0.1629491	0.1841443
**7**	47315	0.1970900	0.0078686	0.1841446	0.2114923
**8**	47315	0.2299153	0.0115178	0.2114930	0.2520045
**9**	47315	0.3038271	0.0494331	0.2520049	0.6839410

Legend: These tables report propensity scores for each decile of rural and urban patients. To assure that common support is achieved, propensity scores of each decile should be similar between rural and urban patients to assure a common support. For example, comparing the highest decile (decile = 9) in rural to highest deciles (decile = 9) in urban, these values should be nearly the same – which is reflected in our data tables above.

© 2021 Greenwood-Ericksen M et al. *JAMA Network Open*.

eTable 5. Marginal Effects with 95% CI of 30-Day Mortality for Urban vs Rural and Non-CAH vs CAH

**How does treatment at an urban ED lower (-) or raise (+) 30-day mortality odds?**			
**Diagnosis**	**Marginal Effects**	**95% CI (%)**	**P-value**
Chest pain	- 0.3%	-0.5, -0.2	<0.000
Urinary Tract Infection	- 0.1%	-0.5, 0.3	0.59
Chronic obstructive pulmonary disease	- 0.1%	-0.5, 0.3	0.74
Cardiac dysrhythmia	+ 0.1%	-0.3, 0.5	0.68
Pneumonia	- 0.1%	-1.9, -0.5	0.001
Congestive heart failure	+ 0.1%	-0.8, 0.9	0.9
Abdominal pain	- 1.0%	-1.3, -0.6	<0.000
Syncope	+ 0.0%	-0.4, 0.3	0.79
Septicemia	+ 0.9%	-0.7, 2.5	0.283
Cerebrovascular accident (e.g. stroke)	- 0.4%	-1.8, 0.1	0.55
GI hemorrhage	- 0.2%	-1.1, 0.7	068
Acute myocardial infarction	+ 0.2%	-1.0, 1.5	0.73
Acute renal failure	-2.4%	-4.4, -0.5	0.013
Intestinal obstruction	- 0.8%	-1.9, 0.3	0.17
Femur fracture	- 0.3%	-1.5, 0.9	0.63
Fluid/electrolyte disturbance	- 1.2%	-1.9, -0.5	0.001
Malaise and fatigue	- 2.0%	-2.7, -1.3	<0.000
Transient ischemic attack	- 0.8%	-1.3, -0.3	0.003
Skin infection	+ 0.1%	-0.3, 0.5	0.72
Dizziness and vertigo	- 0.2%	-0.3, 0.00	0.031
Nausea and vomiting	- 1.0%	-1.6, -0.5	0.000
Spondylosis (e.g., back pain)	- 0.3%	-0.5, 0.0	0.58
Intracranial injury	+ 2.8%	1.1, 4.6	0.02
Biliary tract disease	- 0.2%	-1.1, 0.7	0.71

Legend. This table reports marginal effects in the post-matched cohort, testing the difference in 30-day mortality comparing rural vs. urban. For ease of interpretation within this study's context, rural is the referent. Thus, the marginal effects can be understood as treatment in an urban ED reduces (or raises, if a positive value) the 30-day mortality for a given condition by X% as compared to rural.

© 2021 Greenwood-Ericksen M et al. *JAMA Network Open*.

eFigure 1. Critical Access vs Non–Critical Access Mortality and ED Revisits by Commonly Encountered ED Conditions

Conditions sorted by mortality outcome.

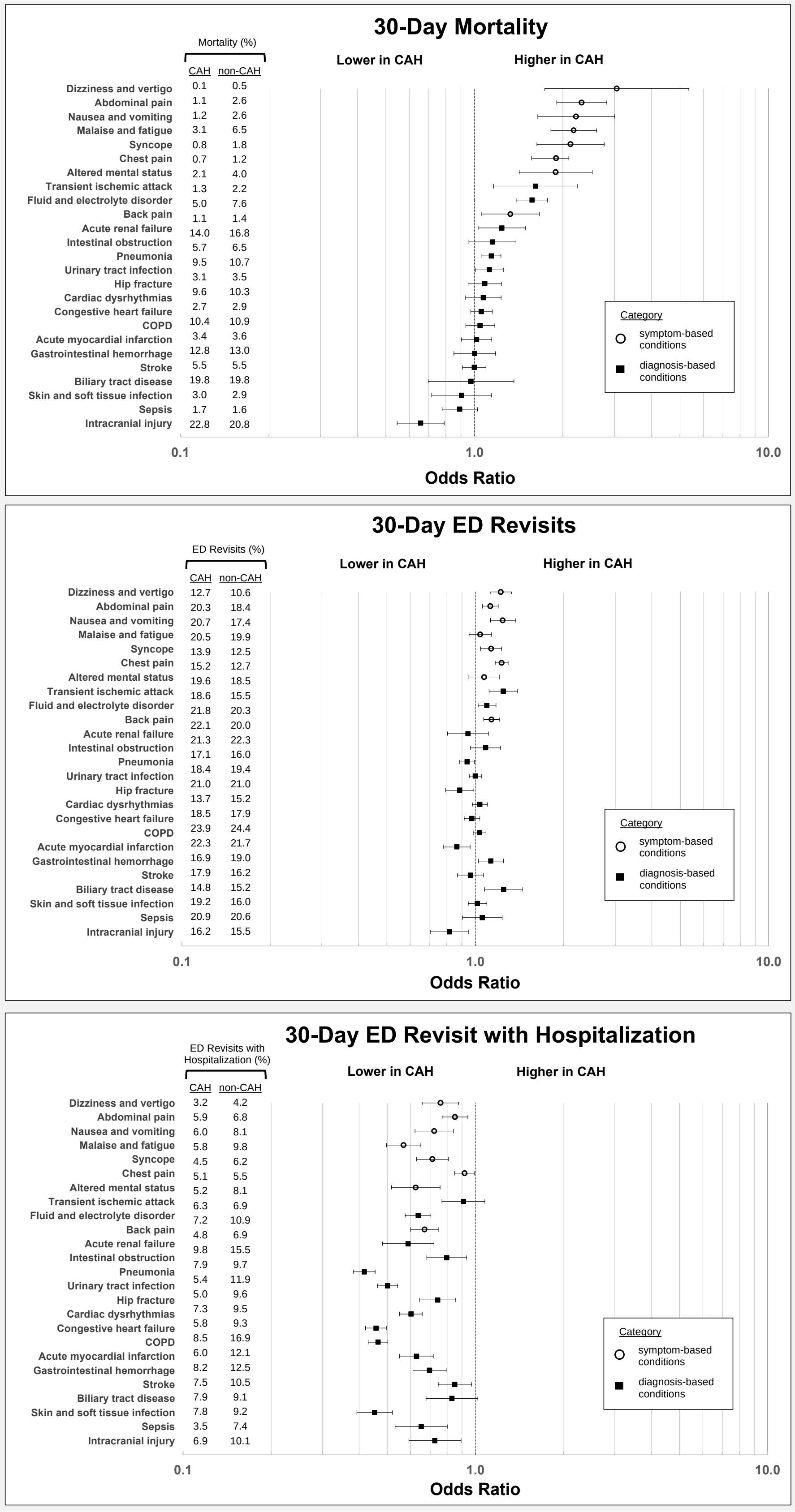

Legend: These three panels represent events occurring after the index ED visit and reflect post-matched results. In the first panel, 30-day mortality is death within 30 days of index ED visit regardless of disposition (hospitalize, transfer, discharge). In the second panel, 30-day ED revisits are those in which the patient returned to the ED within 30 days of an index ED visit which resulted in discharge (so excluding those hospitalized or transferred). The third panel is the same population as the second, but in which the subsequent ED revisit resulted in hospitalization (which includes transfer).

© 2021 Greenwood-Ericksen M et al. *JAMA Network Open*.

eFigure 2. Critical Access vs Non–Critical Access Hospitalization and Transfer Patterns by Commonly Encountered ED Condition

Conditions sorted by mortality outcome as in eFigure 1.

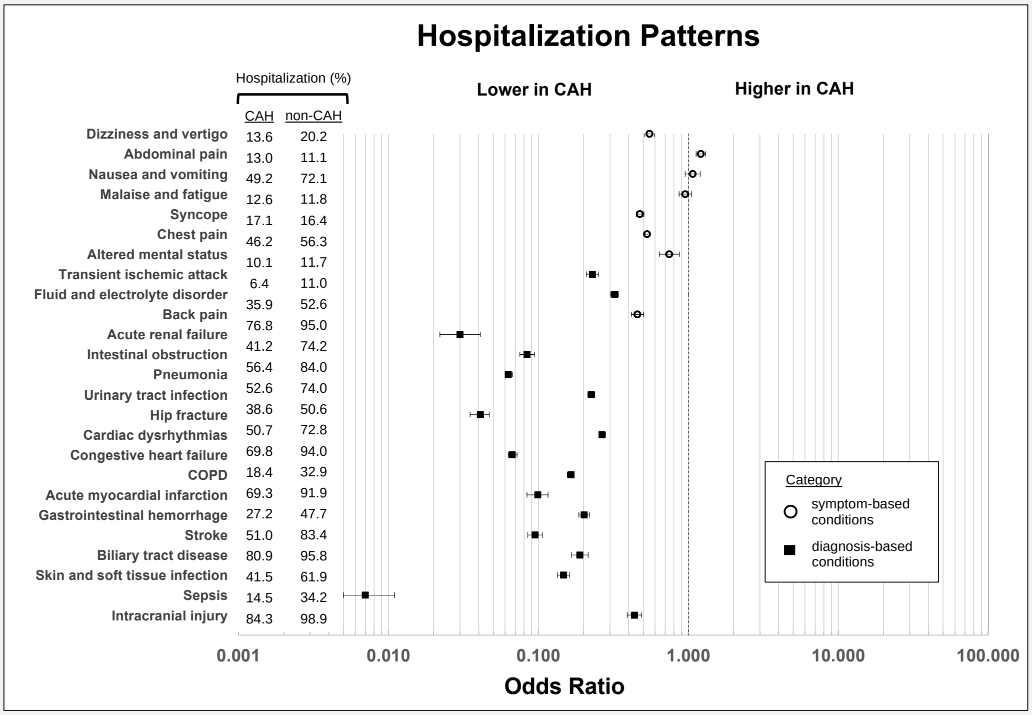

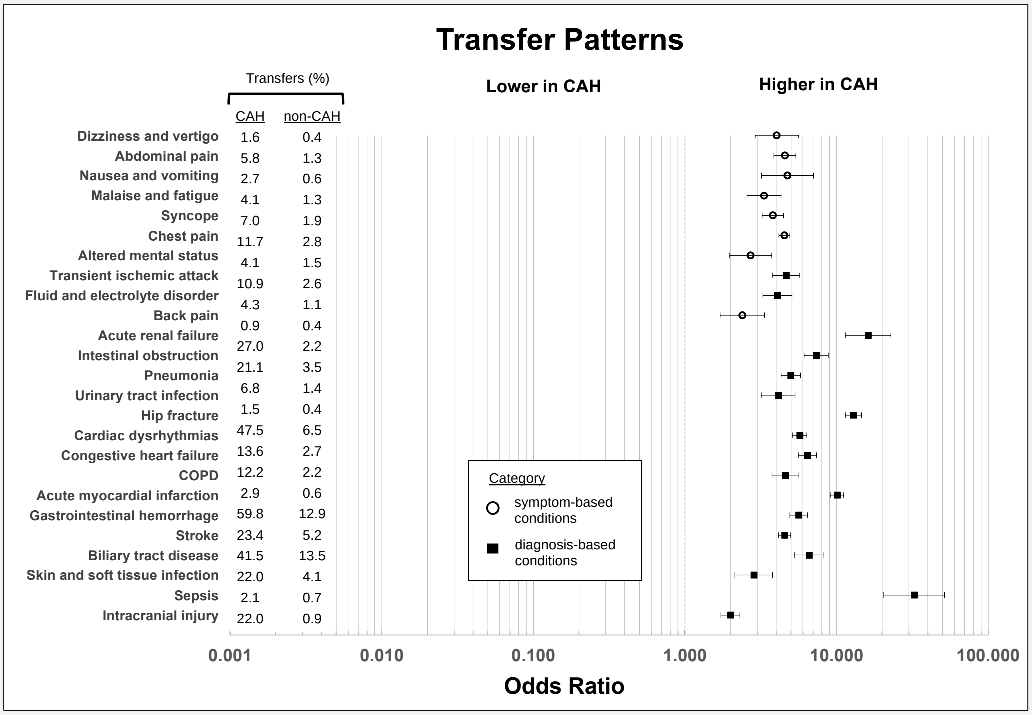

Legend: These two panels represent events occurring for all index ED visits and represent post-matched results. In the first panel, hospitalization is defined as an index visit which resulted in hospitalization or transfer. In the second panel, transfer is defined as the index visit resulting in a disposition of transferred to another acute care hospital. Conditions sorted by mortality outcome from Figure 2. Note different scales on x-axis.

© 2021 Greenwood-Ericksen M et al. *JAMA Network Open*.
